# Haploid like spermatid generation by transplantation of neonatal mouse testicular tissue into the epididymal fat of castrated adult mouse

**DOI:** 10.5935/1518-0557.20240084

**Published:** 2025

**Authors:** Hossein Eyni, Zohreh Mazaheri, Hooman SadriArdekani, Mansoureh Movahedin

**Affiliations:** 1 Department of Anatomical Sciences, Faculty of Medicine, Tarbiat Modares University, Tehran, Iran; 2 Department of Anatomy, Stem Cell and Regenerative Medicine Research Center, School of Medicine, Iran University of Medical Sciences, Tehran, Iran; 3 Basic Medical Sciences Research Center, Histogenotech Company, Tehran, Iran; 4 Wake Forest Institute for Regenerative Medicine, Wake Forest School of Medicine, Winston-Salem, North Carolina, USA; 5 Department of Urology, Wake Forest School of Medicine, Winston-Salem, North Carolina, USA

**Keywords:** spermatid, graft, testicular tissue, epididymal fat

## Abstract

**Objective:**

Many cancer survivors may experience irreversible infertility due to chemotherapy treatment for childhood cancer. In this study, spermatogenesis development was evaluated following the grafting of fresh and frozen-thawed testicular tissue from neonatal mice to the epididymal fat of adult mice.

**Methods:**

After bilateral castration of recipient mice, fresh or frozen-thawed neonatal testis tissues were grafted into the epididymal fat of the mice. Grafted testicular tissue was evaluated eight weeks after implantation using H&E staining, real-time PCR, immunofluorescence staining, and TUNEL assay. Blood was drawn from recipient mice to determine testosterone, FSH, and LH levels.

**Results:**

A gradient of different types of germ cells, from spermatogonia to elongated spermatids was observed. The upregulation of meiotic and post-meiotic genes and proteins in fresh and frozen grafted groups confirmed the progression of meiosis and post-meiosis in grafted tissues. There were no significant differences in the expression of apoptosis and necrosis genes between the grafted and non-grafted control groups. Additionally, no significant differences were observed between the control and experimental groups in hormonal assessments.

**Conclusions:**

The optimal hormonal and temperature conditions of the epididymal fat could support spermatogenesis in grafted immature testicular tissue. This grafting technique could pave the way for fertility preservation.

## INTRODUCTION

For many cancer survivors, chemotherapy and radiation treatments for childhood cancer can result in permanent infertility (Doungkamchan & Orwig, 2021). The current survival rate of 80% among these cancer patients (Gatta *et al*., 2009) supports the higher priority of fertility preservation techniques (Wyns *et al*., 2015; Schover *et al*., 2002; Sadri-Ardekani *et al*., 2013), as long-term infertility becomes a significant issue affecting their quality of life. Grafting stored immature testicular tissue before cancer treatment can potentially become a method of fertility preservation for these individuals (Wyns *et al*., 2010; Wyns *et al*., 2011; Albamonte & Vitullo, 2023).

Due to a lack of niche support, in vitro mammalian spermatogenesis using isolated cells or ex-vivo tissue culture has had limited success (Galdon *et al*., 2016; Ghorbani *et al*., 2019). Initially developed in rodents (Brinster & Zimmermann, 1994; Jiang & Short, 1995), germ cell transplantation to recipient testes has been applied to larger animal models (Honaramooz *et al*., 2003b; Honaramooz *et al*., 2003a; Mikkola *et al*., 2006; Herrid *et al*., 2006; Rodriguez-Sosa *et al*., 2006; Hermann *et al*., 2012). Nevertheless, germ cell transplantation is currently inefficient and technically difficult in large animal models (Mulder *et al*., 2016; Nagano *et al*., 1999; Dobrinski *et al*., 1999, Shetty *et al*., 2020). Only the transplantation of germ cells from rats and hamsters to the mouse testis successfully resulted in complete spermatogenesis (Clouthier *et al*., 1996; Ogawa *et al*., 1999; Zhang *et al*., 2003). Non-rodent donor germ cells could not differentiate beyond the spermatogonial expansion stage in the mouse testis, presumably due to an incompatibility between the donor germ cells and the microenvironment in the mouse testis (Dobrinski *et al*., 2000; Nagano *et al*., 2002). Xenografting of immature equine testicular tissue under the back skin of castrated male immunodeficient mice maintained the environment of testicular tissue and supported the progression through meiosis with the appearance of haploid cells (Shutler *et al*., 1991). Fresh prepubertal testicular tissue survives well when xenografted into the back and testis of shaved mice and can differentiate into mature spermatogenesis, according to studies with nonhuman primates (Honaramooz *et al*., 2004; Ntemou *et al*., 2019b).

After xenografting cryopreserved immature testicular tissue, successful long-term survival and proliferation of human spermatogonia have been reported (Wyns *et al*., 2007; Wyns *et al*., 2008; Goossens *et al*., 2008). These studies could not confirm the functionality of Spermatogonial Stem Cells (SSC) in cryopreserved grafted tissues. In these studies, germ cells were unable to progress beyond the pachytene spermatocyte stage. Two crucial factors in ensuring the success of fertility preservation approaches in childhood cancer survivors are preserving the maximum number of functional SSC in stored testicular tissue and utilizing an optimal transplantation procedure. Consequently, selecting the optimal site for grafting is one of the most fundamental aspects of transplantation. Most studies have transplanted testicular tissue into the subcutaneous layer of the mouse’s back, resulting in the arrest of spermatogenesis in early meiosis (Jahnukainen *et al*., 2007; Wyns *et al*., 2008; Ntemou *et al*., 2019b). It has long been understood that epididymal fat is required for spermatogenesis. The removal of the epididymal fat pad impeded spermatogenesis and increased the concentration of Follicle-Stimulating Hormone (FSH) but had no effect on testosterone production or serum Luteinizing Hormone (LH) concentration (Chu *et al*., 2010; Hansel, 2010). When the surface temperature of the scrotum and the back skin were measured with a Variotherm infrared camera, the mean temperature of the scrotum was 5°C lower than the shaved back skin surface (Luetjens *et al*., 2008). Due to the favorable hormonal and temperature conditions of epididymal fat, we hypothesized that the grafting of neonatal testicular tissue to the epididymal fat area could be an ideal location for spermatogenesis.

This study assessed the development of spermatogenesis after grafting fresh and frozen-thawed neonatal mouse testicular tissue fragments to the epididymal fat region of bilaterally orchiectomized adult mice.

## MATERIAL AND METHODS

### Study design and donor testicular tissue preparation

Six male neonatal (3-5 days old) male Naval Medical Research Institute (NMRI) mice served as donors, while six male adults (6-8 weeks old) NMRI mice served as recipients in the experimental group. Three neonatal and three adult male mice testis tissue were used as control groups. All the animal experiments were carried out per the Institutional Animal Care and Use Committee (IACUC) of Tarbiat Modares University in Tehran, Iran.

**Figure 1:**
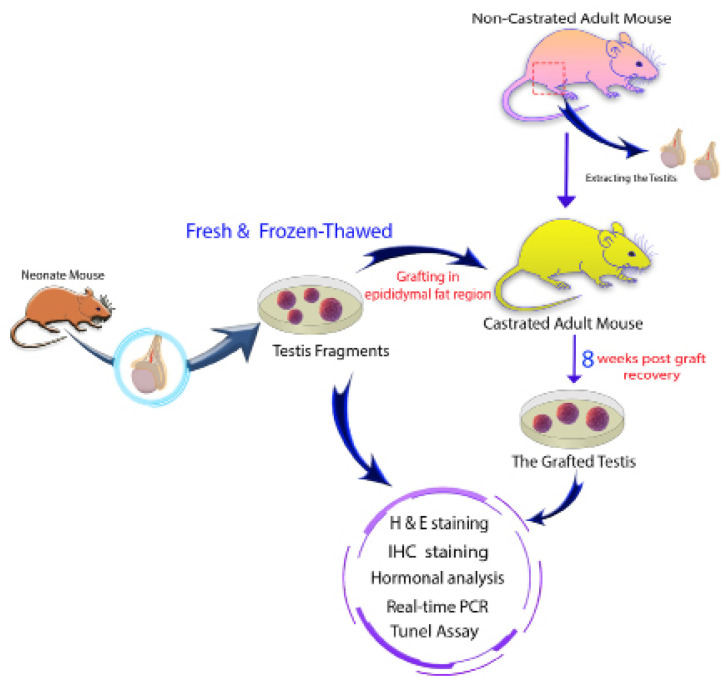
Study design and donor testicular tissue preparation.

Donor mice testes were extracted and transferred immediately to ice-cold Phosphate-Buffered Saline (PBS). The tunica albuginea was eliminated, and the testes were fragmented (approximately 1mm^3^). Testis fragments were stored on ice in Dulbecco’s modified Eagle’s medium (DMEM, USA, Gibco) with 10% Fetal Bovine Serum (FBS, USA). Then, testes fragments were separated into “fresh” and “frozen” fragments. Before grafting, a portion of the fresh fragments of donor testicular tissue was fixed as baseline reference (control) histology (Figure 1).

### Freeze and thawing of neonate testis tissue

Fragments of testicular tissue were equilibrated in freezing media containing DMEM with 5% FBS and 7% glycerol (cat. no. G2025; Sigma, St. Louis, MO, USA); Testis tissue fragments and 0.45 mL of the freezing medium were packaged into 0.5-mL plastic mini straws at room temperature (one testis tissue fragment per straw). Straws were sealed and loaded into a programmable freezer (IceCube 14S; catalog no. 16821/ 2000; Minitube, Ingersoll, ON, Canada) via the slow freezing program described below. The temperatures of the sample and the chamber were monitored throughout the freezing procedure by inserting a thermocouple into one straw and another into the chamber. The freezing program was developed and modified based on a report on the cryopreservation of porcine testis tissue (Abrishami *et al*., 2010).

Initially, straws were maintained at 22°C for 10 min before being cooled to 4°C at a -1°C/min rate, held at 4°C for 5 min, cooled to 0.3°C/min from 4°C to -8°C, held at -8°C for 10 min, cooled 0.5°C/min from -8°C to -50°C, then 10°C/min from -50°C to -90°C, and finally held for 10 min at -90°C. Straws were dipped directly into Liquid Nitrogen (2) at this point and stored until analysis or grafting. Before analysis or grafting, the fragments of cryopreserved testicular tissue were thawed/warmed as follows: Straws containing cryopreserved tissues were removed from the LN2 tanks and immersed in a 37°C water bath until the ice melted (approximately 11 secs). The sealed ends of the straws were cut, and tissues were drained into 2 mL of the first thawing solution (DMEM containing 20% FBS and 0.5 M sucrose) at 37°C and incubated for 1 min. The tissues were then washed in the second solution (DMEM + 20% FBS) at 37°C for 1 to 2 min and stored on ice in this medium until immediate analysis or grafting.

### Grafting of testicular tissue fragments

The recipient mice were anesthetized with an intraperitoneal injection of sterile physiological saline containing a mixture of ketamine (80mg/kg) and xylazine (10mg/kg) (Upjohn Pharma, Germany). A ventral medial incision was made in the recipient’s abdominal skin, and the testes were extracted. Following bilateral orchiectomy, four fragments of fresh or frozen-thawed neonatal testis tissue were grafted into the epididymal fat adjacent to the testicular artery of the recipient. The incisions were closed by an absorbable VICRYL^®^ (Johnson & amp; Johnson Co., USA) suture.

### Histology

After eight weeks, the recipient mice were sacrificed by cervical dislocation. The grafts were removed from the fat and fixed in Bouin’s solution overnight. After washing in 70% ethanol, grafts were embedded in paraffin (for all histological staining) and cut into 5µm sections. Hematoxylin and eosin were used to stain tissue sections (H&E). The non-grafted control neonatal testicular tissues were similarly processed. An AxioPlan microscope (Carl Zeiss GmbH, Germany) equipped with an AxioCam camera was used to evaluate the level of spermatogenesis and seminiferous tubule morphology by observing slides.

### Real-time RT-PCR analysis

Total Ribonucleic Acid (RNA) was extracted from the control (non-grafted) and grafted testicular tissues using QIAzol (Qiagen, Germany) per the manufacturer’s instructions. Afterward, RNA was treated with Deoxyribonuclease (DNase I) (EN0521; Fermentas, Vilnius, Lithuania) to eliminate genomic contamination. Concentrations of RNA were subsequently determined by Ultra Violet (UV) spectrophotometry (Eppendorf, Germany). The cDNAs were synthesized from 500 ng DNase-treated RNA samples with a RevertAid™ First Strand cDNA Synthesis kit (K1622; Fermentas, Germany) using oligo (dT) primers. The gene expression of Promyelocytic Leukemia Zinc Finger (PLZF) (Zbtb16), Tektin 1 (Tekt1), Transition Protein 1 (Tnp1), B Cell Lymphoma-Associated X (Bax), B-cell Lymphoma 2 (Bcl2), Tumor Necrosis Factor Receptor 1 (Tnfr1) and Receptor Interacting Protein Kinase-3 (Rikp3) was analyzed, and Beta-actin (Actb) was used as a housekeeping gene. The primer gene sequences were obtained from the National Center for Biotechnology Information (NCBI) database, and their exon and intron sequences were determined for Polymerase Chain Reaction (PCR) reactions. Primer design was done using the Primer3 online software. The designed primers were blasted to confirm their accuracy and ensure that only the mRNA sequences of genes synthesized by Cinnagen were reproduced (Table 1).

**Table 1:** The list and details of RT-PCR primers that were used to evaluate germ cell development, apoptosis and necrosis.

Gene	Accession Number	Forward Reverse	Product size (bp)
PLZF (Zbtb16)	NM_001033324.2	5’-GCTGCTGTCTCTGTGATGG-3’ 5’-GGGCTGATGGAACATAGGGG-3’	154 bp
Tekt1	NM_053285.1	5’-GCTGGCTGAACATCTGG-3’ 5’-TTCTTGCTGCGTGATGGC-3’	91 bp
Tnp1	NM_003284.3	5’-TGTGATGCGGCAATGAGC-3’ 5’-CGACTGGGATTTACCCACTC-3’	142 bp
Bax	NM_017059.2	5’-TTTGCTACAGGGTTTCATCCAG-3’ 5’-GTCCAGTTCATCGCCAATTC-3’	139 bp
Bcl2	NM_016993.1	5’-GAGAGCGTCAACAGGGAGAT-3’ 5’-ACAGCCAGGAGAAATCAAACA-3’	169 bp
Tnfr1	NM_001065.4	5’-CCTACTTGGTGAGTGACT-3’ 5’-ACCTGGGACATTTCTTTC-3’	134 bp
Rikp3	NM_002415.2	5’-GGAATCAGGGAGATGGAA-3’ 5’-CAGTTGTTGAAGACGAGA-3’	158 bp
Actb	NM_001101	5’-TTACTGAGCTGCGTTTTACAC-3’ 5’-ACAAAGCCATGCCAATGTTG-3’	90 bp

PCRs were performed using Master Mix and SYBR Green I (Cat# S7563, Thermo Fisher) in a StepOne™ thermal cycler (Applied Biosystems, USA). The program started with an initial melting cycle for 5 min at 95°C to activate the polymerase, followed by 40 cycles of melting (30 sec at 95°C), annealing (30 sec at 58°C), and extension (30 sec at 72°C). Melting curve analyses validated the quality of the PCR reactions. Each gene’s efficiency was determined using a standard curve (logarithmic dilution series of cDNA from the testes). Each sample’s reference gene (Actb) and target gene were amplified in the same run. All runs were performed in triplicate. The relative expression of the target genes (before and after the graft of neonatal testis tissue and adult testis tissue) was determined using the CT method and normalized to the housekeeping gene. Adult testis cDNA was used as a positive control.

### Immunofluorescence

In order to evaluate germ cell development, paraffin blocks fixed in Bouin’s solution were cut into 5 µm sections and mounted on glass slides. After deparaffinization and rehydration, slides were washed in PBS. Then, antigen retrieval was conducted in 10 mM sodium citrate/distilled water (pH 6.0) for 30 min in an autoclave at 95 ^°^C. The slides were cooled for 30 min at room temperature. The sections were permeabilized with 0.3% Triton X-100 for 30 min (Sigma, USA), and non-specific binding sites were blocked by incubating in 10% goat serum in PBS for 30 min. Afterward, slides were incubated with primary antibody PLZF (ab189849, Abcam, Cambridge, MA, USA), Synaptonemal Complex Protein 3 (SYCP3) (ab97672, Abcam, Cambridge, MA, USA), and Acrosin Binding Protein (ACRBP) (ab211145, Abcam, Cambridge, MA, USA) diluted 1:200 in PBS overnight at 4°C. After three washes in PBS, samples were incubated with Alexa Fluor 488 Donkey Anti-Mouse Immunoglobulin G (IgG) at a 1:500 dilution (Abcam, USA) at 37^◦^C for 1 h. Nucleus staining was performed using 4’,6-Diamidino-2-Phenylindole (DAPI, Sigma). Prepared slides were observed under an inverted fluorescence microscope (Nikon TE 2000, Japan).

### TUNEL assay

Apoptotic cells in controls (non-grafted) and grafted tissues were detected by Terminal deoxynucleotidyl transferase (TdT) dUTP Nick-End Labeling (TUNEL) assay. The sections were stained using a Roche kit per the manufacturer’s instructions. Initially, fixed slides were deparaffinized, dehydrated, and permeabilized with 15µg/ml proteinase K for 30 minutes at 37˚C (Roche, Germany). TUNEL reaction mixture was then added to slides. The sections were allowed to incubate for 1 h at 37˚C. After three PBS washes, the sections were incubated for 30 min at 37˚C with Converter-POD. DAPI was employed for nucleus staining. Prepared slides were observed under an inverted fluorescence microscope (Nikon TE 2000, Japan).

### Endocrine assessment

For hormonal analysis, the adult recipient mice were divided into four groups (non-grafted control, bilateral castrated control, fresh-grafted, and frozen-thawed grafted). Eight weeks after grafting, all four groups obtained serums from the mice’s blood samples taken during anesthesia. Each mouse’s serum sample was analyzed using a commercial kit to detect testosterone (LS-F10019, USA), FSH (LS-F9659, USA), and LH (LS-F22503, USA).

### Statistical analysis

Data were expressed as mean±standard deviation. The Statistical Package for the Social Sciences (SPSS) software (v. 18, SPSS Inc., USA) was used to compare data via Analysis of Variance (ANOVA) and independent sample t-tests. *p* values<0.05 were deemed statistically significant.

## RESULTS

### Survival of grafted tissue

**Figure 2:**
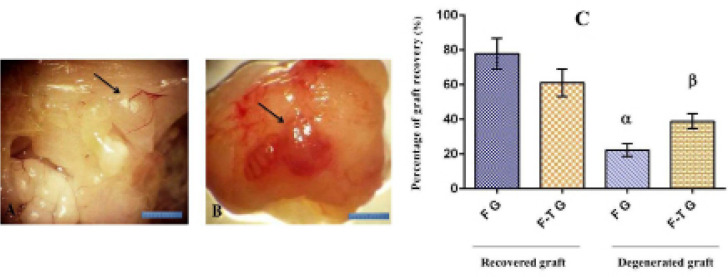
Morphological study and graft recovery rate of fresh and frozen-thawed grafted testis tissue in the epididymal fat region. A: Fresh grafted testis tissue from a neonate (arrow) in the epididymal fat of an adult mouse (8 weeks after graft). B: Grafted frozen-thawed neonatal testis tissue (arrow) in mature mouse epididymal fat (8 weeks after graft). C: At eight weeks post-transplantation, there was no significant difference between the percentage of recovered grafts harvested from fresh and frozen-thawed tissues. (F G: Fresh grafted; F-T G: Frozen-thawed). *α, β:* Significant difference compared to other groups. Scale bar: 10mm.

Survival and growth of the grafted tissue were very apparent in the recipient mice’s epididymal fat. Figure 2 shows a typical example of the epididymal fat with surviving fresh (2A) and frozen-thawed (2B) testis tissue grafts. Blood supply formation within the grafted tissues was evident (Figure 2). Eight weeks after transplantation, 77.8% (14/18) of the fresh grafts and 61.11% (11/18) of the frozen-thawed grafts were recovered. Graft recovery is defined as the detectable graft collected eight weeks after transplantation. This difference was statistically insignificant between the fresh and frozen-thawed groups (Figure 2C).

### Histological assessment of spermatogenesis in grafted tissue

**Figure 3:**
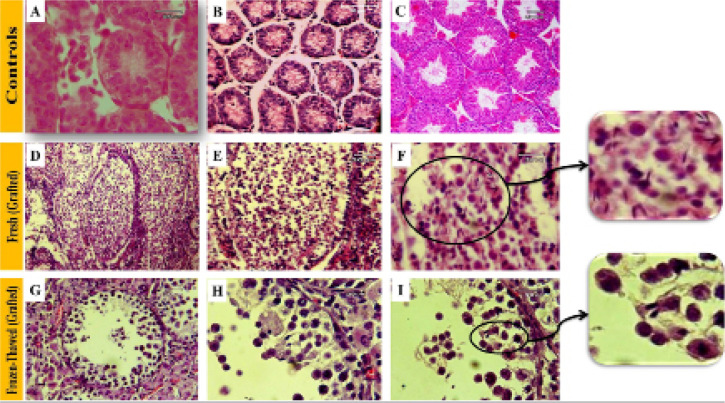
Histological characteristics of fresh and frozen-thawed testis grafts in the epididymal fat region. Fresh and frozen-thawed mouse neonatal testis tissue stained with hematoxylin and eosin (H&E) before and after grafting. A: Fresh neonate testis tissue (before graft). B: Frozen-thawed neonate testis tissue (before graft). C: Adult testis tissue. D-F: Fresh grafted testis tissue (8 weeks after graft). G-I: Frozenthawed grafted testis tissues (n8 weeks after graft). The insert depicts the area with elongated spermatids.

At the time of grafting, the seminiferous tubules of donor mice’s testicular tissue contained only two cell types: spermatogonia, the only germ cell type, and Sertoli cells (Figure 3A, B). Figure 3 illustrates the common morphology of grafts (fresh and frozen-thawed grafts). The seminiferous tubules in the fresh and frozen-thawed groups exhibited variable levels of spermatogenic activity and contained various cell types, ranging from undifferentiated spermatogonia to elongated spermatid, compared with the mature testis as a control. All the grafts showed some degree of spermatogenesis, as indicated by at least one seminiferous tubule with developing germ cells.

### Gene expression analyses of grafted testis tissues

**Figure 4:**
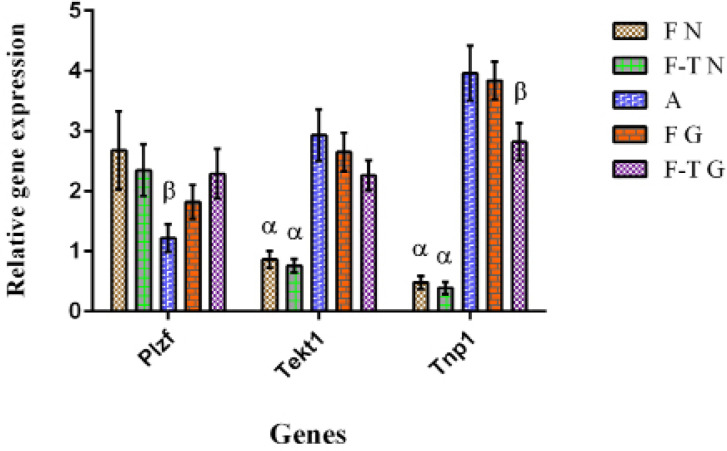
Preand post-meiotic germ cell development. Analysis of spermatogenesis gene expression using real-time PCR. F N: Fresh neonate, F-T N: Frozen-thawed neonate, A: Adult, F G: Fresh grafted, and F-T G: Frozen-thawed grafted. The levels of mRNA were normalized relative to Actb as an internal control. The mean expression values (± SEM, n=3; *p*<0.05) are depicted by bar graphs. α, β: Significant difference with other groups in the same gene.

The expression of testis development, apoptosis, and necrosis-related genes were determined. Expression of PLZF, a marker for undifferentiated spermatogonial cells, was significantly lower in the adult control group than in other groups, including grafted and control (non-grafted) neonatal testes tissues (Figure 3). The meiotic (Tekt1) and post-meiotic (Tnp1) genes were upregulated in both the fresh and frozen-thawed grafted groups, and they were significantly different from the fresh and frozen-thawed non-grafted neonatal control groups (Figure 4).

**Figure 5:**
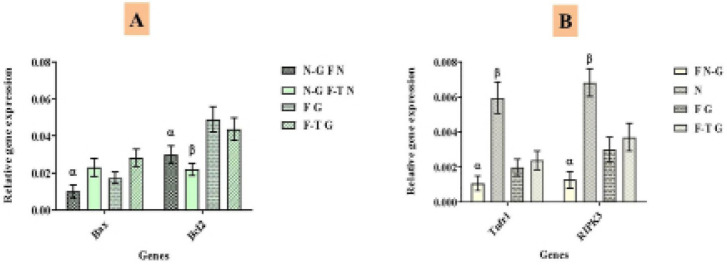
Evaluation of cell apoptosis and necrosis in the epididymal fat region grafted testis tissues. A) Real-time PCR analysis of apoptosis gene expression. N-G F N: Non-grafted fresh neonate, F-T N: Non-grafted fresh neonate; F G: Fresh grafted; and F-T G: Frozen-thawed grafted. B) Real-time PCR analysis of necrosis gene expression. F N-G: Fresh non-grafted, N: Necrosis model (induced torsion*), F G: Fresh grafted, and F-T G: Frozen-thawed grafted. mRNA levels were normalized relative to Actb as an internal control. Histograms display the mean expression values (± SEM, n=3; *p*<0.05). α, β: Significant difference between this gene and other groups. *: Testicular torsion was accomplished via a midline, ventral incision. Each testis was exposed through the incision, and the gubernaculum and the avascular epididymal-testicular membrane were incised. The testis was rotated at 720° for 2 hours.

The Bax gene expression as an apoptosis marker was significantly lower in the fresh neonatal group (non-grafted) compared to other groups. The expression of the Bcl2 gene as an anti-apoptosis marker was considerably lower in the frozen-thawed neonatal group (non-grafted) than in the other groups (Figure 5A).

The expression of the Tnfr1 and Rikp3 genes as necrosis markers was analyzed in four groups: fresh non-grafted necrosis model (induced torsion), fresh grafted, and frozen-thawed grafted. The expression of necrosis markers in the frozen-thawed neonatal grafted group was not significantly higher than in the non-grafted and fresh grafted groups (Figure 5B).

### Immunofluorescence

**Figure 6:**
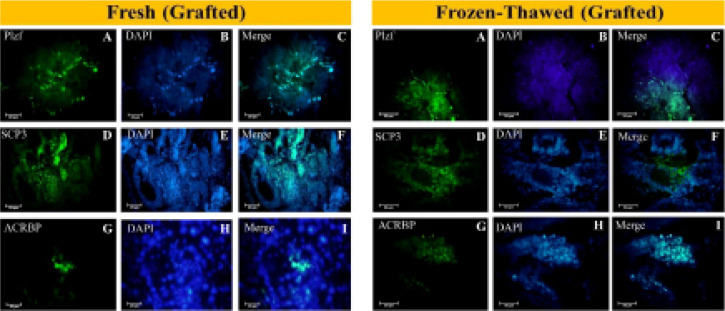
The fresh non-grafted neonatal and adult testis tissues were used as a control.

**Figure 7:**
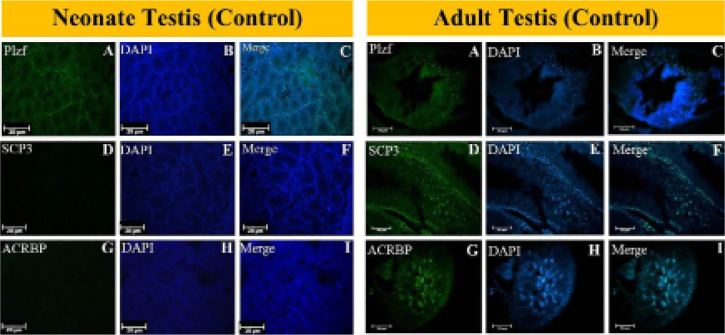
Different types of germ cells in grafted fresh and frozen-thawed neonatal testis tissues in the epididymal fat region. Immunostaining fresh and frozen-thawed grafted testis tissue groups for PLZF (Zbtb16), SCP3, and ACRBP. A-C: PLZF, D-F: SCP3 and G-I: ACRBP. Nuclei were stained with DAPI.

The expression of spermatogenesis development stage-specific markers, the PLZF (undifferentiated spermatogonial cell marker), SCP3 (spermatocyte marker), and ACRBP (elongated spermatid marker) proteins, were analyzed by immunofluorescence staining eight weeks after grafting. The fresh non-grafted neonatal and adult testis tissues were used as a control (Figure 6). The PLZF marker was expressed in fresh non-grafted neonatal, adult testis, and grafted testis tissues, whereas SCP3 and ACRBP were only expressed in adult and grafted testis tissues (Figure 7).

### Apoptosis assay

**Figure 8:**
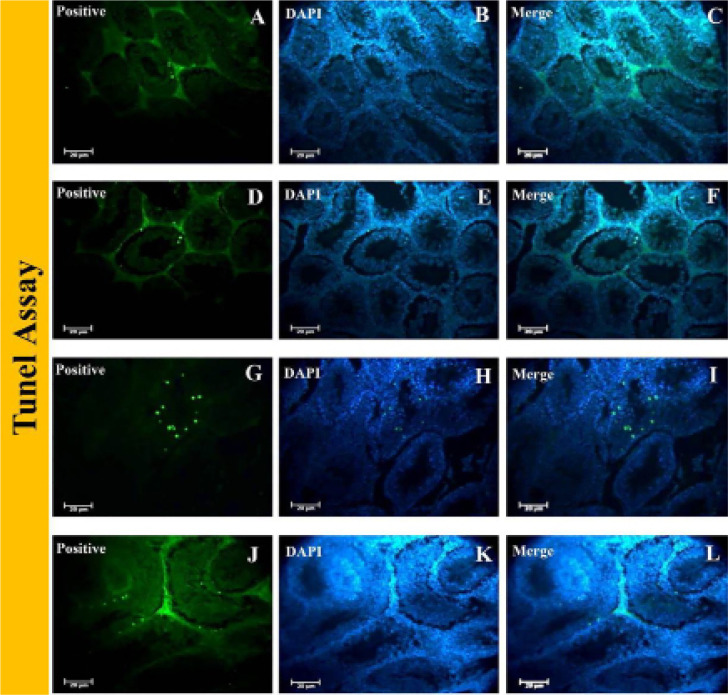
DNA integrity of grafted testis tissues in the epididymal fat region. TUNEL assays of control and experimental groups. A-C: Fresh non-grafted neonate testis tissue, D-F: Fresh grafted neonate testis tissue, G-I: Frozen-thawed non-grafted neonate testis tissue, and J-L: Frozen-thawed grafted neonate testis tissue. Nuclei were stained with DAPI.

TUNEL assay was used to detect DNA fragmentation and determine the level of apoptosis in grafted tissue. The non-grafted fresh and frozen-thawed neonatal testis tissues served as controls. The frequency of seminiferous tubules with apoptotic cells and the number of apoptotic cells per seminiferous tubule were greater in the grafted groups than in the control (non-grafted) groups and the frozen-thawed groups than in the fresh groups (Figure 8).

### Hormonal evaluation

**Figure 9:**
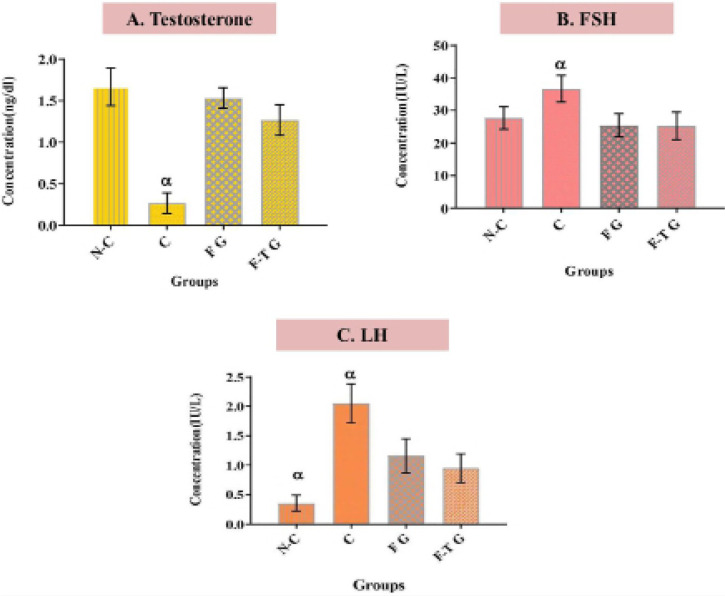
Serum Testosterone, FSH, and LH Levels. N-C: Non-castrated adult mouse, C: Castrated adult mouse, F G: Fresh grafted, and F-T G: Frozen-thawed grafted. Histograms show mean expression values (± SEM, n=3; *p*<0.05). α: Significant difference with other groups in the same hormone.

Measuring the total testosterone level in the mouse blood serum of the grafted and non-castrated groups revealed the highest amount, whereas the castrated group’s testosterone level was extremely low (Figure 9A). Significantly higher serum FSH levels were found in the castrated group compared to other groups (Figure 9B). The intact group had significantly lower serum LH levels than other groups (Figure 9C). In addition, a tendency for high LH serum levels in the castrated group, low LH serum levels in the intact group, and moderate LH serum levels in the grafted groups were observed.

## DISCUSSION

Long-term infertility is a significant side effect of numerous cancer treatments, as both radiotherapy and chemotherapy have deleterious effects on the differentiating spermatogonia (Howell & Shalet, 2001; Krassas & Pontikides, 2005; Kanbar *et al*., 2022). Although adult cancer patients can preserve their sperm before beginning treatment, prepubescent boys cannot because their spermatogenesis has not yet begun. Therefore, new fertility preservation methods must be developed for prepubescent boys requiring gonadotoxic treatment. Preserving and transplantation of testicular tissue may be a promising cancer treatment strategy (Honaramooz, 2023). Testicular tissue grafting without vascular anastomosis is contingent on developing new vascular blood vessels to support spermatogenesis activity and reduce ischemic injury (Ntemou *et al*., 2019b). It has been demonstrated that spermatogenesis is enhanced and apoptosis is decreased in grafted testicular tissue by employing various techniques, such as the use of angiogenesis-affecting factors, such as growth factors (Schmidt *et al*., 2006; Ntemou *et al*., 2019a), hydrogels, or tissue engineering scaffolds (Poels *et al*., 2016). Due to adequate blood supply, apoptosis and necrosis were not significantly different between the grafting and non-grafting groups in the present study. The graft site is essential to the regeneration process, which results in the growth of grafted tissue and the improvement of spermatogenesis. In general, researchers believe that transplantation to orthotopic sites is more successful than transplantation to heterotopic sites. Orthotopic transplantation allows for a more accurate assessment of spermatid development and function in their natural environment (Jiang *et al*., 2021).

In this approach, spermatid precursors are transplanted into the seminiferous tubules of the testes, where they can develop and differentiate in a physiological context (Hanson *et al*., 2021). Also, orthotopic transplantation is more likely to preserve the integrity of the blood-testis barrier (BTB), a critical component of the testicular microenvironment that regulates the exchange of molecules between the bloodstream and the seminiferous tubules (Faraldo *et al*., 2022). Finally, orthotopic transplantation enables the study of spermatid development in a more dynamic and responsive system (Tomiyama *et al*., 2024). Due to certain these advantages, such as easier access to heterotopic sites (Luetjens *et al*., 2008), attempts to improve methods utilizing this location have continued. The benefit of orthotopic grafting is that the testicular tissue is transferred into its natural environment, including high levels of testosterone, and is not exposed to hyperthermic conditions, as is the case with subcutaneous grafting. A meiotic arrest was observed in ectopic marmoset testicular grafts, while complete spermatogenesis was observed in scrotal grafts (Luetjens *et al*., 2008). After gonadotoxic treatment with follicular development and restoration of ovulatory cycles, intra-ovarian transplantation has been performed in females (Donnez *et al*., 2004). Luetjens *et al*. (2008) compared the under-scrotal (ectopic transplantation site) skin surface temperature to the deep testicular temperature (orthotopic transplantation site). They found a 5°C temperature difference between the two locations. It has been demonstrated that the scrotal surface (30°C) and testis (35°C) are cooler than the ectopic transplantation site on the backs of the animals (Luetjens *et al*., 2008). This may inhibit the differentiation of germ cells. The higher temperature at the site of ectopic transplantation on the animal’s back may contribute to the arrest of germ cell development. Multiple species (Jia *et al*., 2007; Schwalm *et al*., 2007) have reported this effect on the maturation of germ cells, and it was also used as an experimental method for male contraception. Therefore, numerous factors contribute to the survival of grafted testicular tissue and the resumption of spermatogenesis, with temperature and hormonal conditions at the grafting site being the most crucial. As a graft site in our study, epididymal fat was utilized. Epididymal fat was deemed suitable for transplantation and supporting spermatogenesis with the production of special agent(s) in its place (Hansel, 2010; Chu *et al*., 2010) and having a lower body temperature than other areas. Our findings indicate that testis grafting into epididymal fat, as described here, yields optimal survival and functionality of the graft.

In this study, we evaluated the development of spermatogenesis eight weeks after grafting fresh and frozen-thawed fragments of neonatal mouse testicular tissue to the epididymal fat region of bilaterally castrated adult mice. Because the spermatogenesis duration in mice is approximately 34.5 days, we want to have a complete spermatogenesis in grafted tissues. For this reason, we selected a time more than 34.5 days for more certainly. Our findings demonstrated that neonatal mouse testis tissue can survive transplantation into the epididymal fat of castrated adult mice and that spermatogenesis up to the level of elongated spermatids can be observed eight weeks later. In the current study, an increase in the size and diameter of seminiferous tubules and an improvement and resumption of spermatogenesis were observed in the fresh and frozen-thawed groups based on H&E staining results. We observed that the grafted tissue generated an adequate blood supply, with optimal growth and differentiation of germ cells in seminiferous tubules. In previous studies, an increase in the size and diameter of the seminiferous tubules was observed with the growth and differentiation of germ cells up to the spermatocyte stage; however, meiosis was not achieved. Jahnukainen *et al*. reported sperm production when prepubescent monkey testis fragments were autologously transplanted into adult castrated host animals (Jahnukainen *et al*., 2012; Yamini *et al*., 2016). Van Saen *et al*. found that four months after transplanting fresh and frozen-thawed human immature testis tissue into the back skin of immunodeficient mice, no meiosis occurred in the prepubertal grafted tissues (Van Saen *et al*., 2011). In our study, there was no significant difference in the expression of the PLZF gene between grafted and control groups. After grafting, Tekt1 and Tnp1 expression as meiotic and post-meiotic markers are comparable to that of the adult control group. The expression of apoptosis and necrosis markers in the grafted groups did not differ significantly from that of the control groups. Using H&E staining and immunofluorescence, the presence of SYCP3 and ACRBP cells in grafted seminiferous tubules confirmed the progression of germ cell differentiation. The expression of PLZF as an undifferentiated spermatogonial cell marker stayed stable post-grafting. The TUNEL technique was used in our study to assess the amount of cell apoptosis in grafted and control groups. We found that the number of apoptotic cells was higher in the frozen-thawed group than in the fresh group. Previous research has also shown that the frozen-thawed group had higher cell apoptosis than the fresh group (Gholami *et al*., 2013). This study showed that serum testosterone levels returned to normal after grafting fresh or frozen-thawed tissue into castrated recipient animals.

According to the findings of this study, spermatogenesis development, including the growth and differentiation of germ cells, was observed in the grafted testis tissue of neonatal mice to the epididymal fat of adult castrated mice. This study demonstrated a substantial increase in spermatogenesis compared to ectopic grafting sites. In future preclinical studies aimed at preserving the fertility of childhood cancer survivors, epididymal fat may be utilized as a suitable site for transplantation and induction of spermatogenesis.

## ABBREVIATIONS

Promyelocytic Leukemia Zinc Finger: PLZF
Spermatogonial Stem Cells: SSC
Follicle-Stimulating Hormone: FSH
Luteinizing Hormone: LH
Naval Medical Research Institute: NMRI
Institutional Animal Care and Use Committee: IACUC
Phosphate-Buffered Saline: PBS
Dulbecco’s modified Eagle’s medium: DMEM
Fetal Bovine Serum: FBS
Liquid Nitrogen: LN2
Hematoxylin and Eosin: H&E
Ribonucleic Acid: RNA
Deoxyribonuclease: DNase
Ultra Violet: UV
Tektin 1: Tekt1
Transition Protein 1: Tnp1
B Cell Lymphoma-Associated X: Bax
B-cell lymphoma 2: Bcl2
Tumor Necrosis Factor Receptor 1: Tnfr1
Receptor Interacting Protein Kinase-3: Rikp3
Beta-actin: Actb
Polymerase Chain Reaction: PCR
National Center for Biotechnology Information: NCBI
Synaptonemal Complex Protein 3: SYCP3
Acrosin Binding Protein: ACRBP
Immunoglobulin G: IgG
4’, 6- Diamidino-2-Phenylindole: DAPI
Terminal deoxynucleotidyl transferase (TdT) dUTP Nick-End Labeling: TUNEL
Testosterone: T
ANalysis Of Variance: ANOVA
Statistical Package for Social Sciences: SPSS
